# EKF-SIRD model algorithm for predicting the coronavirus (COVID-19) spreading dynamics

**DOI:** 10.1038/s41598-022-16496-6

**Published:** 2022-08-04

**Authors:** Abdennour Sebbagh, Sihem Kechida

**Affiliations:** Laboratoire d’Automatique et Informatique de Guelma (LAIG), Université 8 mai 1945 Guelma, Bp: 401, 24000 Guelma, Algeria

**Keywords:** Biomedical engineering, Computational science, Biological techniques

## Abstract

In this paper, we study the Covid 19 disease profile in the Algerian territory since February 25, 2020 to February 13, 2021. The idea is to develop a decision support system allowing public health decision and policy-makers to have future statistics (the daily prediction of parameters) of the pandemic; and also encourage citizens for conducting health protocols. Many studies applied traditional epidemic models or machine learning models to forecast the evolution of coronavirus epidemic, but the use of such models alone to make the prediction will be less precise. For this purpose, we assume that the spread of the coronavirus is a moving target described by an epidemic model. On the basis of a SIRD model (Susceptible-Infection-Recovery- Death), we applied the EKF algorithm to predict daily all parameters. These predicted parameters will be much beneficial to hospital managers for updating the available means of hospitalization (beds, oxygen concentrator, etc.) in order to reduce the mortality rate and the infected. Simulations carried out reveal that the EKF seems to be more efficient according to the obtained results.

## Introduction

Since its appearance in late December 2019, the new COVID-19 epidemic has spread rapidly across the world. The first cases of COVID-19 were reported in Wuhan, China and this disease then dilated to Europe, North and South America affecting the most of developed countries such as Italy, France, USA, etc. where sporadic cases have been imported via returning travelers from China.

While it has long seemed spared, or almost, by Covid-19, the African continent is not immune to this coronavirus epidemic, it is now affected like the rest of the world, even if the number of deaths remains very limited. A sudden acceleration in the number of cases was observed in July and August and then the contaminations slowed down again. At the start of the year, we are witnessing a "new wave" very visible in the North of the continent, and observable in several large countries of the East and the South, while the health authorities are getting organized for the arrival of the first doses of vaccine.

As of February 13, 2021, the virus had spread to most African countries, with more than 3 734 227 confirmed cases and more than 97 863 reported deaths, including Algeria with 112 461 cases and 2 970 deaths^1^.

For the purpose of control and prevention from the spread of this outbreak of coronavirus, Algerian authorities have implemented various containment measures since March 28, 2020, including traffic restrictions, contact tracing, mandatory face masks in public spaces, entry or exit screening, quarantine and awareness campaigns.

The current outbreak of coronavirus disease (COVID-19) is declared as Public Health Emergency of International concern and a pandemic by the World Health Organization (WHO). This alarming situation has prompted scientists to indulge in studies concerning the transmission dynamics and forecasting of the virus to the most affected countries in the world such as Chine^2,3^, Italy^4^, France^5^, India^6^ and then to other countries^7–13^, etc.

These works focus on epidemiological studies whose the main objective is to develop strategies to fight against the spread of the coronavirus and provide guidance to control its transmission dynamic^14,15^. A considerable number of strategies require or involve mathematical models dedicated for studying infection diseases such as SIRD, SIR or SEIR models in different context^16–18^ (analysis, forecasting the spread and prediction).

The most of these works are intended to the modeling of transmission dynamics with the aim to predict the trend of the epidemic and control the outbreak evolution. In this context, authors of^19–23^ proposed mathematical models translating the transmission dynamics of COVID-19 to forecast the number of active cases or to estimate the total number of infected and deaths^7^, while those of^5^ develop a strategy based on SIR model to estimate the actual number of people infected and to deduce the IFR (Infected Fatality Ratio). The forecast of future COVID-19 cases has discussed in^24^ using regression analysis.

The estimation of infection, mortality and recovery rates and the basic reproduction number ($$R$$_0_) are provided in^3^ using a SIRD model. Afterwards, Dhillon and all study the trend analysis of mortality and recovery rate considering scenario of most affected countries and Indian States^6^. To mitigate disease transmission, mathematical models introducing a quarantine measures are formulated by Liu and all in^2^ and Mandal and all in^25^. Other research works establish the prediction of epidemic peak under the impact of lockdown using an improvised compartment mathematical model^26^ (SEIR or SEIRD) i.e., Susceptible ($$S$$)-Exposed ($$E$$)-Infected ($$I)$$-Recovered ($$R$$)-Death ($$D$$) while, in^27,28^, authors study the forecast of the spread tendency of the COVID-19 through an improved SEIR model. Others methods such as fractional concept, optimization algorithms, Artificial Neural Network… are introduced sometimes for study the growth of cumulative confirmed and cured people and sometimes for formulate the prediction problem as an optimization framework^29^ or to estimate the COVID-19 cases^8^.

Additionally, and for containing the epidemic spread in African countries, research works are being conducted in the top infected countries through studies modeling and forecasting of COVID-19. Among which, there have been some comparative studies between the African countries including Algeria^30–32^.

However, there are a little peer reviewed papers about epidemiological profile in Algerian territory; these research studies consider traditional epidemic models (SIR, SI, SEIR, …) dedicated to historical data analysis for forecasting the incidence and /or estimation of parameters^33–37^.

In all these developed methodologies, the authors consider mathematical models whose parameters are estimated over a limited period of time. The model once defined is applied in different studies of COVID'19 evolution without taking into account the update of the model parameters and the various measures taken by those responsible.

In this work, we project the engineering techniques used in targets tracking on epidemiology assuming that the spread of the coronavirus is a moving target described by an epidemic model. The idea is to investigate the Kalman filter on SIRD model with the goal to predict the spreading of the Covid 19 and to effectively manage the burden of COVID-19 pandemic in Algeria.

This study shows the disease profile in the Algerian territory since February 25, 2020 to February 13, 2021. Here we are fascinated in applying the extended Kalman filter (EKF) using an epidemic SIRD model to provide a daily prediction of infection, mortality and recovery rates and the basic reproduction number (R_0_).

In addition, these data are much beneficial to hospital managers and public health decision-makers for updating the available means of hospitalization (beds, oxygen concentrator, etc.) in order to reduce the mortality rate and the infected.

The rest of this paper is organized in 4 sections. “Problem formulation” Section is dedicated the problem formulation and the description of chosen model. The next section details Bayesian approach and more precisely the EKF algorithm used in the context of this work. The application of this technique and the simulation results are discussed in “Simulation results” section and finally, the last section recapitulates concluding remarks of this study and to suggest some outlooks for future works.

## Problem formulation

In the literature, the works carried out in the epidemiology study use mathematical models each stratify the dynamics of individuals. The choice of these individuals depends on the problem formulation. The most of the epidemic models for human-to-human transmission rely on the susceptible-infected-recovered (SIR) structure, considered as a fundamental model widely used to delineate various infectious diseases. SIRD model is the standard famous SIR model incorporating an additional compartment: Death class (D). Other structures have emerged to monitor the dynamics of others compartments (classes), such as quarantined susceptible individuals, asymptomatic infectious individuals, isolated infected individuals, exposed individuals, etc.^3,21,22,26,37^

For the SIRD model, the population $N$ is divided into sub-population: susceptible $(S)$, infected $(I)$, recovered $(R)$ and deceased $(D)$ for all time $k$, i.e., $N=S+I+R+D$.

**Figure 1:**
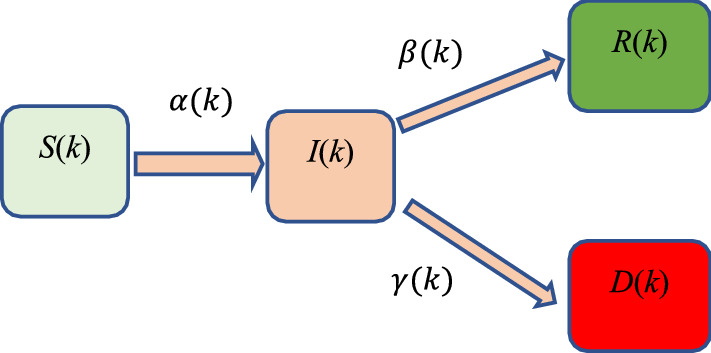
Flow between the populations of SIRD model.

The discrete nonlinear SIRD model is given by:

1 $$S\left( {k + 1} \right) = S\left( k \right) - \frac{\alpha \left( k \right)}{N}S\left( k \right)I\left( k \right)$$

2 $$I\left( {k + 1} \right) = I\left( k \right) + \frac{\alpha \left( k \right)}{N}S\left( k \right)I\left( k \right) - \beta \left( k \right)I\left( k \right) - \gamma \left( k \right)I\left( k \right)$$

3 $$R\left( {k + 1} \right) = R\left( k \right) + \beta \left( k \right)I\left( k \right)$$

4 $$D\left( {k + 1} \right) = D\left( k \right) + \gamma \left( k \right)I\left( k \right)$$

where $\alpha \left(k\right)$, $\beta \left(k\right)$ and $\gamma (k)$ are the daily infection, daily recovery and daily death rates respectively, see Fig. 1, note that, these rates are optimized daily using the least square method (LSM) as follows:

If we accept that $S=N,$ then:

5 $$\alpha \left( k \right) = \frac{{\mathop \sum \nolimits_{j = 1}^{k} I\left( j \right) \cdot \Delta S\left( j \right)}}{{\mathop \sum \nolimits_{j = 1}^{k} I^{2} \left( j \right)}}$$

If $$S\ne N,$$ then:

6 $$\alpha \left( k \right) = N \cdot \frac{{\mathop \sum \nolimits_{j = 1}^{k} S\left( j \right) \cdot I\left( j \right) \cdot \Delta S\left( j \right)}}{{\mathop \sum \nolimits_{j = 1}^{k} I^{2} \left( j \right) \cdot S^{2} \left( j \right)}}$$

7 $$\beta \left( k \right) = \frac{{\mathop \sum \nolimits_{j = 1}^{k} I\left( j \right) \cdot \Delta R\left( j \right)}}{{\mathop \sum \nolimits_{j = 1}^{k} I^{2} \left( j \right)}}$$

8 $$\gamma \left( k \right) = \frac{{\mathop \sum \nolimits_{j = 1}^{k} I\left( j \right) \cdot \Delta D\left( j \right)}}{{\mathop \sum \nolimits_{j = 1}^{k} I^{2} \left( j \right)}}$$

$I(j)$ is the total currently infected in the time $j$ (day).

$\Delta S\left(j\right)=S\left(j\right)-S(j-1)$ is daily new coronavirus cases at time $j$

$\Delta R(j)$=$R\left(j\right)-R\left(j-1\right)$ is daily new recovered at time $j$

$$j)$$ =$$D\left(j\right)-D(j-1)$$ is daily new deceased at time $$j$$

We suppose that:

$$x_{1} \left( k \right) = S\left( k \right),~x_{2} \left( k \right) = I\left( k \right),~x_{3} \left( k \right) = R\left( k \right),\,{\text{and}}\,x_{4} \left( k \right) = D\left( k \right)$$

Then the SIRD model becomes:

9 $$\left( {\begin{array}{*{20}c} {x_{1} \left( {k + 1} \right)} \\ {x_{2} \left( {k + 1} \right)} \\ {\begin{array}{*{20}c} {x_{3} \left( {k + 1} \right)} \\ {x_{4} \left( {k + 1} \right)} \\ \end{array} } \\ \end{array} } \right) = \left( {\begin{array}{*{20}c} {f_{1} \left( {X_{k} } \right)} \\ {f_{2} \left( {X_{k} } \right)} \\ {\begin{array}{*{20}c} {f_{3} \left( {X_{k} } \right)} \\ {f_{4} \left( {X_{k} } \right)} \\ \end{array} } \\ \end{array} } \right) = \left( {\begin{array}{*{20}c} {x_{1} \left( k \right) - \frac{\alpha \left( k \right)}{N}x_{1} \left( k \right) \cdot x_{2} \left( k \right)} \\ {x_{2} \left( k \right) + \frac{\alpha \left( k \right)}{N}x_{1} \left( k \right) \cdot x_{2} \left( k \right) - \beta \left( k \right)x_{2} \left( k \right) - \gamma \left( k \right)x_{2} \left( k \right)} \\ {\begin{array}{*{20}c} {x_{3} \left( k \right) + \beta \left( k \right)x_{2} \left( k \right)} \\ {x_{4} \left( k \right) + \gamma \left( k \right)x_{2} \left( k \right)} \\ \end{array} } \\ \end{array} } \right) + V_{k}$$

${X}_{k}$ is the state vector including susceptible $(S)$, infected $(I)$, recovered $(R)$ and deceased $(D)$, defined as:

$${X}_{k}={\left(\begin{array}{cc}\begin{array}{cc}S& I\end{array}& \begin{array}{cc}R& D\end{array}\end{array}\right)}^{T}$$

${V}_{k}$ is a zero-mean white noise with covariance ${Q}_{V}$.

The Jacobian matrice of this model is obtained as:

10 $$F\left( {X_{k} } \right) = \frac{\partial f}{{\partial X_{k} }} = \left( {\begin{array}{*{20}c} {1 - \frac{{\hat{\alpha }\left( k \right)}}{N}x_{2} \left( k \right)} & { - \frac{{\hat{\alpha }\left( k \right)}}{N}x_{1} \left( k \right)} & {\begin{array}{*{20}c} 0 & 0 \\ \end{array} } \\ {\frac{{\hat{\alpha }\left( k \right)}}{N}x_{2} \left( k \right)} & {1 - \hat{\beta }\left( k \right) - \hat{\gamma }\left( k \right) + \frac{{\hat{\alpha }\left( k \right)}}{N}x_{1} \left( k \right)} & {\begin{array}{*{20}c} 0 & 0 \\ \end{array} } \\ {\begin{array}{*{20}c} 0 \\ 0 \\ \end{array} } & {\begin{array}{*{20}c} {\hat{\beta }\left( k \right)} \\ {\hat{\gamma }\left( k \right)} \\ \end{array} } & {\begin{array}{*{20}c} {\begin{array}{*{20}c} 1 \\ 0 \\ \end{array} } & {\begin{array}{*{20}c} 0 \\ 1 \\ \end{array} } \\ \end{array} } \\ \end{array} } \right)$$

where $\widehat{\alpha }\left(k\right)$, $\widehat{\beta }\left(k\right)$ and $\widehat{\gamma }(k)$ are the predicted daily infection, predicted daily recovery and predicted daily death rates respectively and are calculated as:

11 $$\begin{gathered} \hat{\alpha }\left( k \right) = \frac{{\text{predicted daily new cases}}}{{\text{estmate of total currently infected}}} \hfill \\ = \frac{{\left[ {x_{2} \left( {k + 1/k} \right) - x_{2} \left( k \right)} \right] + \left[ {x_{3} \left( {k + 1/k} \right) - x_{3} \left( k \right)} \right] + \left[ {x_{4} \left( {k + 1/k} \right) - x_{4} \left( k \right)} \right]}}{{x_{2} \left( k \right)}} \hfill \\ \end{gathered}$$

12 $$\hat{\beta }\left( k \right) = \frac{{\text{predicted daily new recovered}}}{{\text{estimate of total currently infected}}} = \frac{{\left[ {x_{3} \left( {k + 1/k} \right) - x_{3} \left( k \right)} \right]}}{{x_{2} \left( k \right)}}$$

13 $$\hat{\gamma }\left( k \right) = \frac{{\text{predicted daily new deceased }}}{{\text{estimate of total currently infected}}} = \frac{{\left[ {x_{4} \left( {k + 1/k} \right) - x_{4} \left( k \right)} \right]}}{{x_{2} \left( k \right)}}$$

The predicted daily new cases = the predicted daily new currently infected + the predicted daily new recovered + the predicted daily new deceased.

We suppose that the measurement equation is given daily by:

14 $$\begin{gathered} Y_{k + 1} = \left( {\begin{array}{*{20}c} {y_{1} \left( {k + 1} \right)} \\ {y_{2} \left( {k + 1} \right)} \\ {y_{3} \left( {k + 1} \right)} \\ \end{array} } \right) = \left( {\begin{array}{*{20}c} {I\left( {k + 1} \right)} \\ {R\left( {k + 1} \right)} \\ {D\left( {k + 1} \right)} \\ \end{array} } \right) = \left( {\begin{array}{*{20}c} 0 & 1 & {\begin{array}{*{20}c} 0 & 0 \\ \end{array} } \\ 0 & 0 & {\begin{array}{*{20}c} 1 & 0 \\ \end{array} } \\ 0 & 0 & {\begin{array}{*{20}c} 0 & 1 \\ \end{array} } \\ \end{array} } \right)\left( {\begin{array}{*{20}c} {x_{1} \left( {k + 1} \right)} \\ {x_{2} \left( {k + 1} \right)} \\ {\begin{array}{*{20}c} {x_{3} \left( {k + 1} \right)} \\ {x_{4} \left( {k + 1} \right)} \\ \end{array} } \\ \end{array} } \right) + W_{k} \hfill \\ Y_{k + 1} = CX_{k + 1} + W_{k} \hfill \\ \end{gathered}$$

with

$${\varvec{C}} = \left( {\begin{array}{*{20}c} 0 & 1 & {\begin{array}{*{20}c} 0 & 0 \\ \end{array} } \\ 0 & 0 & {\begin{array}{*{20}c} 1 & 0 \\ \end{array} } \\ 0 & 0 & {\begin{array}{*{20}c} 0 & 1 \\ \end{array} } \\ \end{array} } \right)$$

${W}_{k}$ is a a zero-mean white noise with covariance ${\sum }_{W}$

## Bayesian filtering

In Bayesian approach we attempt to construct the posterior PDF of the state given all measurements. All available information is used to form such PDF. So, this PDF represents complete solution.

Let ${X}_{k}$, $k\in {\mathbb{N}}$, be the state sequence:

15 $$X_{k} = f_{k} \left( {X_{k - 1} , u_{k - 1} , V_{k - 1} } \right)$$

where ${f}_{k}$ is in generally nonlinear function of the previous state ${X}_{k-1}\in {\mathbb{R}}^{{n}_{x}}$, ${V}_{k-1}\in {\mathbb{N}}^{{n}_{v}}$ is state noise, ${u}_{k-1}\in {\mathbb{R}}^{{n}_{u}}$ is known input, ${n}_{x}$, ${n}_{v}$ et ${n}_{u}$ are dimensions of the state, process and input noise vectors.

let ${Y}_{k}$ be the measurement:

16 $$Y_{k} = h_{k} \left( {X_{k} ,W_{k} } \right)$$

where ${Y}_{k}\in {\mathbb{R}}^{{n}_{y}}$, ${h}_{k}$ is in generally non-linear measurements function, ${W}_{k}\in {\mathbb{N}}^{{n}_{w}}$ is measurement noise, ${n}_{y}$ and ${n}_{w}$ are dimensions of the measurement and measurement noise vectors.

We want to find estimate of the ${X}_{k}$ based on all available measurements at time $k$ (marked as ${Y}_{1:k}$) by constructing the posterior PDF $p({X}_{k}, {Y}_{1:k}).$ It is assumed, that initial PDF $p\left({X}_{0}|{Y}_{0}\right)\equiv p({X}_{0})$ is available. Posterior PDF can be obtained recursively in two stages, namely prediction and update. Suppose that required PDF $p({X}_{k-1}|{Y}_{1:k-1})$ at time step $k-1$ is available. Then using the system model, it is possible to obtain the prior PDF of the state at the time step $k$^38,39^:

17 $$p\left( {X_{k} {|}Y_{1:k - 1} } \right) = \smallint p\left( {X_{k} {|}X_{k - 1} } \right)p\left( {X_{k - 1} {|}Y_{1:k - 1} } \right)dX_{k - 1}$$

Prediction step usually deforms, spreads state PDF due to noise. Measurement $Y_{k}$ is available at time step $k$, so it can be used to update the prior. Using Bayes’ rule, we obtain:

18 $$p\left( {X_{k} {|}Y_{1:k} } \right) = \frac{{p\left( {Y_{k} {|}X_{k} } \right)p\left( {X_{k} {|}Y_{1:k - 1} } \right)}}{{p\left( {Y_{k} {|}Y_{1:k - 1} } \right)}}$$

where the normalizing constant is:

19 $$p\left( {Y_{k} {|}Y_{1:k - 1} } \right) = \smallint p\left( {Y_{k} {|}X_{k} } \right)p(X_{k} |Y_{1:k - 1} )dX_{k}$$

In the update Eq. (19), the measurement ${Y}_{k}$ is used to modify the predicted prior from the previous time step to obtain PDF of the state. Equations (17) and (18) theoretically allow optimal Bayesian solution. But it is only conceptual solution and integrals in these equations are intractable. Solution exists in some restricted cases such as Kalman Filter.

### Kalman filter

Kalman filter together with its basic variants are commonly the used tools in statistical signal processing, especially in the context of causal, real-time applications.

There are several approaches in the derivation of the Kalman Filter. We can assume Gaussian distribution of the deriving process and of the initial state. In the next phase, we derive the posterior distribution of the states given the observations, taking the mean of the resulting distributions as the estimation of the state. The second approach combines a recursive weighted least-squares method with special weighting of the previous estimate of the states in the role of additional measurements^40,41^.

Kalman Filter can be used in estimation of the state ${X}_{k}\in {\mathbb{R}}^{{n}_{x}}$ where posterior PDF is Gaussian in every time step. But in many cases this PDF is not Gaussian and we need to use different approach such as extended Kalman Filter. This method is also labelled as sub-optimal algorithm^42,43^.

### Extended Kalman filter

Most processes in real life are unfortunately nonlinear, and therefore needs to be linearized before they can be estimated by Kalman filter.

The extended Kalman filter (EKF)^38,39,44–46^, is the nonlinear genre of the Kalman filter^41,42^ which linearizes about an estimate of the current mean and covariance^43,47^. The state transition and measurement models for the extended Kalman filter are taken as:

20 $$X\left( {k + 1} \right) = f\left( {X\left( k \right)} \right) + V\left( k \right)$$

21 $$Y\left( {k + 1} \right) = h\left( {X\left( {k + 1} \right)} \right) + W\left( k \right)$$

where $V(k)$ is the process noise with zero mean and covariance ${Q}_{k}$, and $W(k)$ is the measurement noise with zero mean and covariance ${\sum }_{k}$.

The functions $f\left(X\left(k\right)\right)$ and $h\left(X\left(k+1\right)\right)$ are used to compute the predicted state from the previous estimate and predicted measurement from the predicted state, respectively. Instead of applying $f\left(X\left(k\right)\right)$ and $h\left(X\left(k+1\right)\right)$ to the covariance directly, a Jacobian matrix is applied which is evaluated with current predicted states at each time step. Extended Kalman Filter is based upon approximation of the Bayes’ rule using linearization.

Discrete-time extended Kalman filter’s prediction (time update) and correction (measurement update) equations are given by,

- ***Prediction (time update)***

Predict stage can be described using following equations:

22 $$\hat{X}_{k + 1|k} = f\left( {\hat{X}\left( {k|k} \right)} \right)$$

where $\hat{X}_{k + 1|k}$ is the predicted state estimate at time $k + 1$ given measurements up to time $k$ and

23 $$P_{k + 1|k} = \hat{F}_{k} P_{k + 1|k} \hat{F}_{k}^{T} + Q_{k}$$

where ${P}_{k+1|k}$ is the error covariance matrix.

- ***Correction (measurement update)***

Update stage can be described with the following equations:

24 $$\tilde{y}_{k + 1} = Y_{k + 1} - h\left( {\hat{X}_{k + 1|k} } \right)$$

where $\tilde{y}_{k + 1}$ is innovation term,

25 $$S_{k + 1} = \hat{H}_{k + 1} P_{k + 1|k} \hat{H}_{k + 1}^{T} + \sum_{k}$$

where $S_{k + 1}$ is the innovation covariance,

26 $$K_{k + 1} = P_{k + 1|k} \hat{H}_{k + 1}^{T} S_{k + 1}^{ - 1}$$

where $K_{k + 1}$ is the Kalman gain,

27 $$\hat{X}_{k + 1|k + 1} = \hat{X}_{k + 1|k} + K_{k + 1} \tilde{y}_{k + 1}$$

is update state estimate and

28 $$P_{k + 1|k + 1} = \left( {I - K_{k + 1} \hat{H}_{k + 1} } \right)P_{k + 1|k}$$

is update estimate covariance.

Where the Jacobian for state transition and measurement matrices are defined as:

29 $$\hat{F}_{k} = \left. {\frac{\partial f}{{\partial X_{ } }}} \right|_{{X_{k|k} }}$$

30 $$\hat{H}_{k + 1} = \left. {\frac{\partial h}{{\partial X_{ } }}} \right|_{{X_{k + 1|k} }}$$

**Figure 2:**
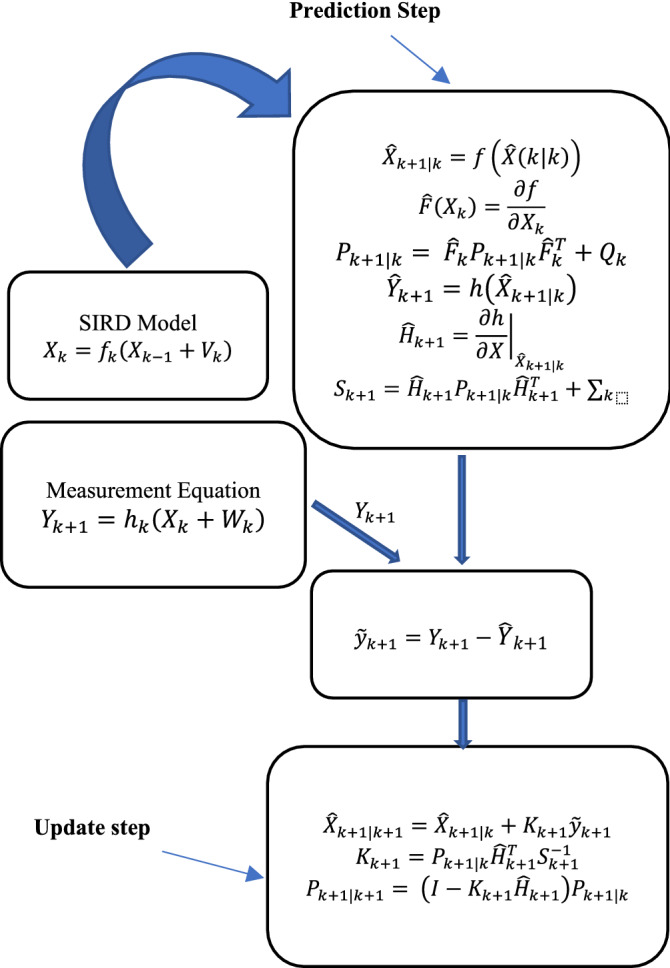
EKF-SIRD Model Algorithm.

Figure 2 Shows the EKF-SIRD Algorithm.

## Simulation results

For the application of EKF estimator on coronavirus (covid-19) modelled by the SIRD model, we use the real data provided by the Ministry of Algerian health and the WHO, from February 25, 2020 to February 13, 2021 in our daily predictions.

We consider that the spread of coronavirus is a target that begins its movement from the initial vector:

$$X\left( 1 \right) = \left( {\begin{array}{*{20}c} {N - 1} & 1 & {\begin{array}{*{20}c} 0 & 0 \\ \end{array} } \\ \end{array} } \right)^{T}$$

where $N = 44219385$ is the Algerian population number. The mean vector and covariance matrice initialization of the EKF according to a Gaussian law are:

$$\hat{X}\left( 1 \right) = \left( {\begin{array}{*{20}c} {N - 1} & 1 & {\begin{array}{*{20}c} 0 & 0 \\ \end{array} } \\ \end{array} } \right)^{T}$$

$$P\left( {1{\text{|}}1} \right) = \left( {\begin{array}{*{20}c} {100} & 0 & 0 & 0 \\ 0 & {100} & 0 & 0 \\ 0 & 0 & {100} & 0 \\ 0 & 0 & 0 & {100} \\ \end{array} } \right)$$

The process noise is zero mean, white and with covariance

$$Q = \left( {\begin{array}{*{20}c} {100} & 0 & 0 & 0 \\ 0 & {100} & 0 & 0 \\ 0 & 0 & {100} & 0 \\ 0 & 0 & 0 & {100} \\ \end{array} } \right)$$

The measurement noise is also zero mean, white, independent of the process noise, and with covariance

$$\sum _{k} = \left( {\begin{array}{*{20}c} {10^{{ - 2}} } & 0 & 0 \\ 0 & {10^{{ - 2}} } & 0 \\ 0 & 0 & {10^{{ - 2}} } \\ \end{array} } \right)$$

**Figure 3:**
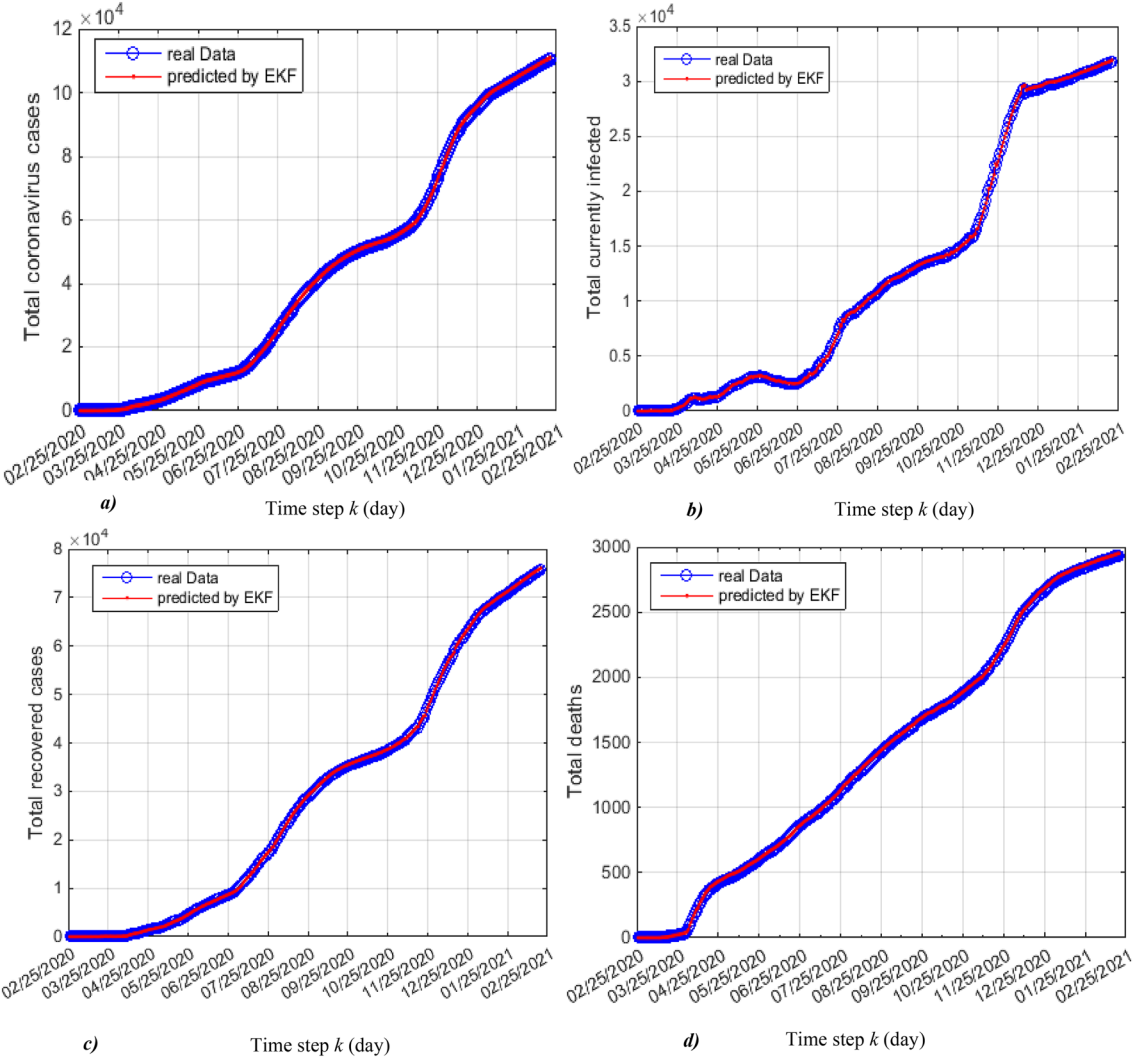
Real and predicted trajectories, (**a**) total coronavirus cases, (**b**) total currently infected, (**c**) total recovered cases and (**d**) total deceased.

The trajectories plotted in Fig. 3a, b, c and d are the real data of Algeria and predicted by EKF of total coronavirus cases, total currently infected, total recovered and total deceased respectively.

We observe that the predicted trajectories by the EKF are superposable on the trajectories of real data, which allowed us to say that the EKF is correctly predicted the evolution of these quantities.

**Figure 4:**
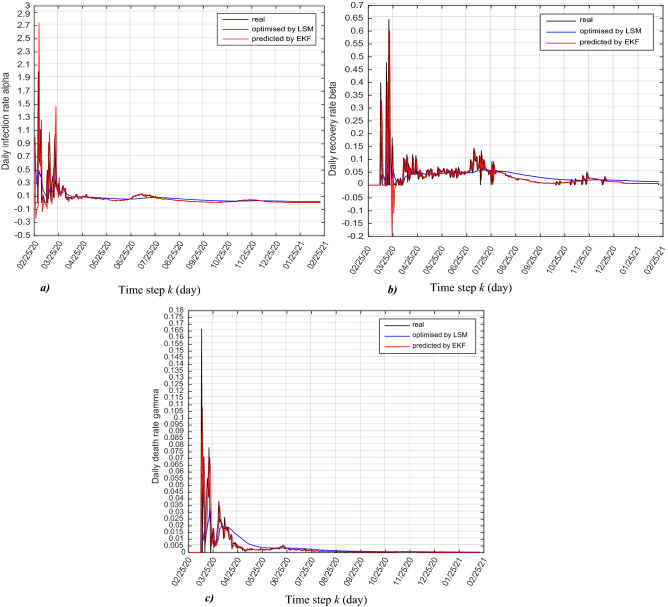
Real and predicted trajectories, (**a**) daily infection rate $\alpha (k)$, (**b**) daily recovery rate $\beta \left(k\right)$ and (**c**) daily death rate $\gamma (k)$.

The daily infection rate $\alpha (k)$, daily recovery rate $\beta (k)$ and daily death rate $\gamma (k)$ are optimised by using least square method (LSM) according to Eqs. (5), (6), (7) and (8), and also predicted by the EKF according to Eqs. (11), (12) and (13) as shown in Fig. 4a, b and c.

**Figure 5:**
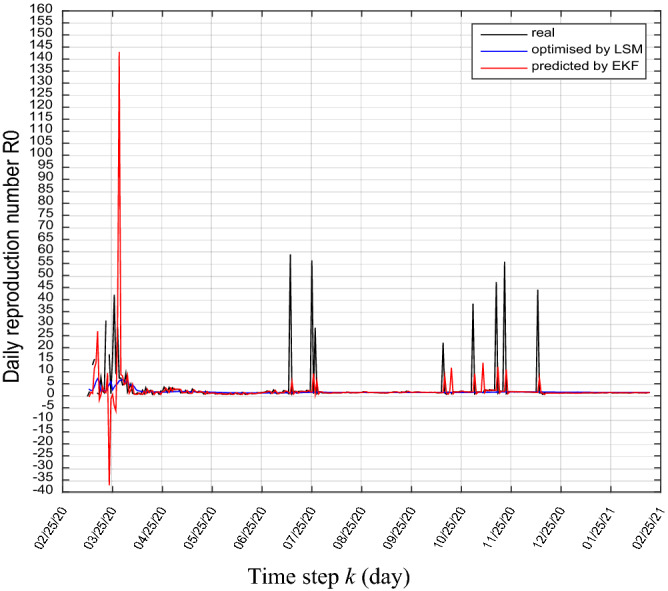
Real and predicted trajectories of daily basic reproduction number ${R}_{0}$.

**Figure 6:**
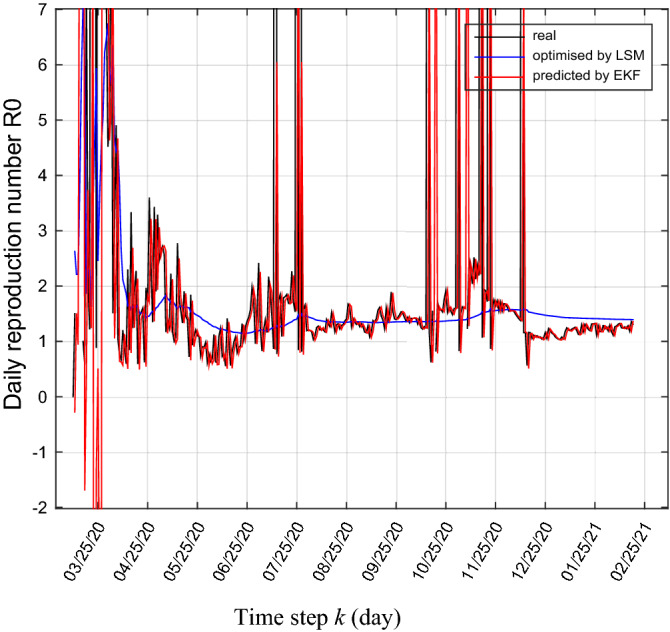
Real and predicted trajectories of daily basic reproduction number ${R}_{0}$(zoom).

From these previous predictions (by LMS and by EKF), we can daily predict the basic reproduction number ${R}_{0}$ as shown in Figs. 5 and 6, according to Eq. (31).

31 $$R_{0} \left( k \right) = \frac{\alpha \left( k \right)}{{\beta \left( k \right) + \gamma \left( k \right)}}$$

We see that generally, the value of the basic reproduction number between the end of April, 2020 and October 15, 2020 is between 1 and 2 except for some disturbances in July, this comes down to the containments measures taken by the country officials (lockdown), including traffic restrictions, contact tracing, mandatory face masks in public spaces.

From the mid of October, 2020 until the end of December, we see some disturbances in the basic reproduction number because of the appearance of the second coronavirus wave, where the daily new coronavirus number has been increased and reached 1133 cases on November 24, 2020.

From the beginning of January, 2021 until February 13, 2021 the basic reproduction number stabilizes between 1 and 1.5.

**Figure 7:**
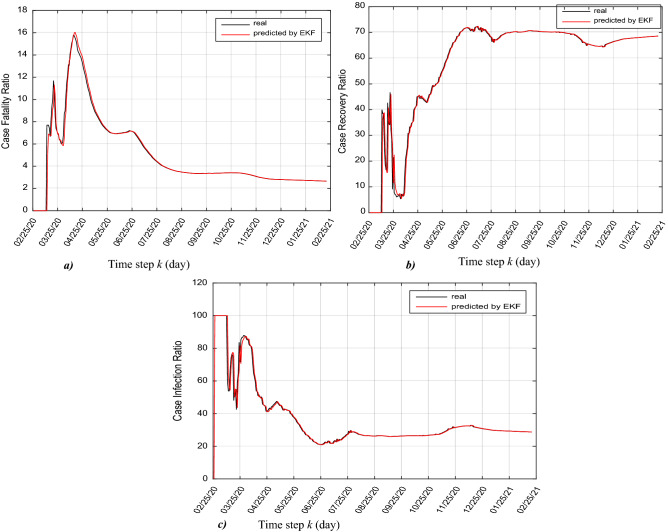
Real and predicted trajectories, (**a**) case fatality ratio, (**b**) case recovery ration and, (**c**) case infection ratio.

Using the predictions by EKF of total currently infected, total recovered and total deceased, we can daily predict the case fatality ratio (CFR), case recovery ratio (CRR) and also case infection ratio (CIR) as shown in Fig. 7a, b and c, according to these equations:

32 $$CFR\left( {k + 1} \right)\% = \frac{{Total\,deceased \left( {x_{4} \left( {k + 1/k} \right) = D\left( {k + 1} \right)} \right)}}{{Total\,coronavirus\,cases = (x_{2} \left( {k + 1/k} \right) + x_{3} \left( {k + 1/k} \right) + x_{4} \left( {k + 1/k} \right))}} \cdot 100$$

33 $$CRR\left( {k + 1} \right)\% = \frac{{Total\,recovered \left( {x_{3} \left( {k + 1/k} \right) = R\left( {k + 1} \right)} \right)}}{{Total\,coronavirus\,cases = (x_{2} \left( {k + 1/k} \right) + x_{3} \left( {k + 1/k} \right) + x_{4} \left( {k + 1/k} \right))}} \cdot 100$$

34 $$CIR\left( {k + 1} \right)\% = \frac{{Total\,currently\,infected \left( {x_{2} \left( {k + 1/k} \right) = I\left( {k + 1} \right)} \right)}}{{Total\,coronavirus\,cases = (x_{2} \left( {k + 1/k} \right) + x_{3} \left( {k + 1/k} \right) + x_{4} \left( {k + 1/k} \right))}} \cdot 100$$

**Figure 8:**
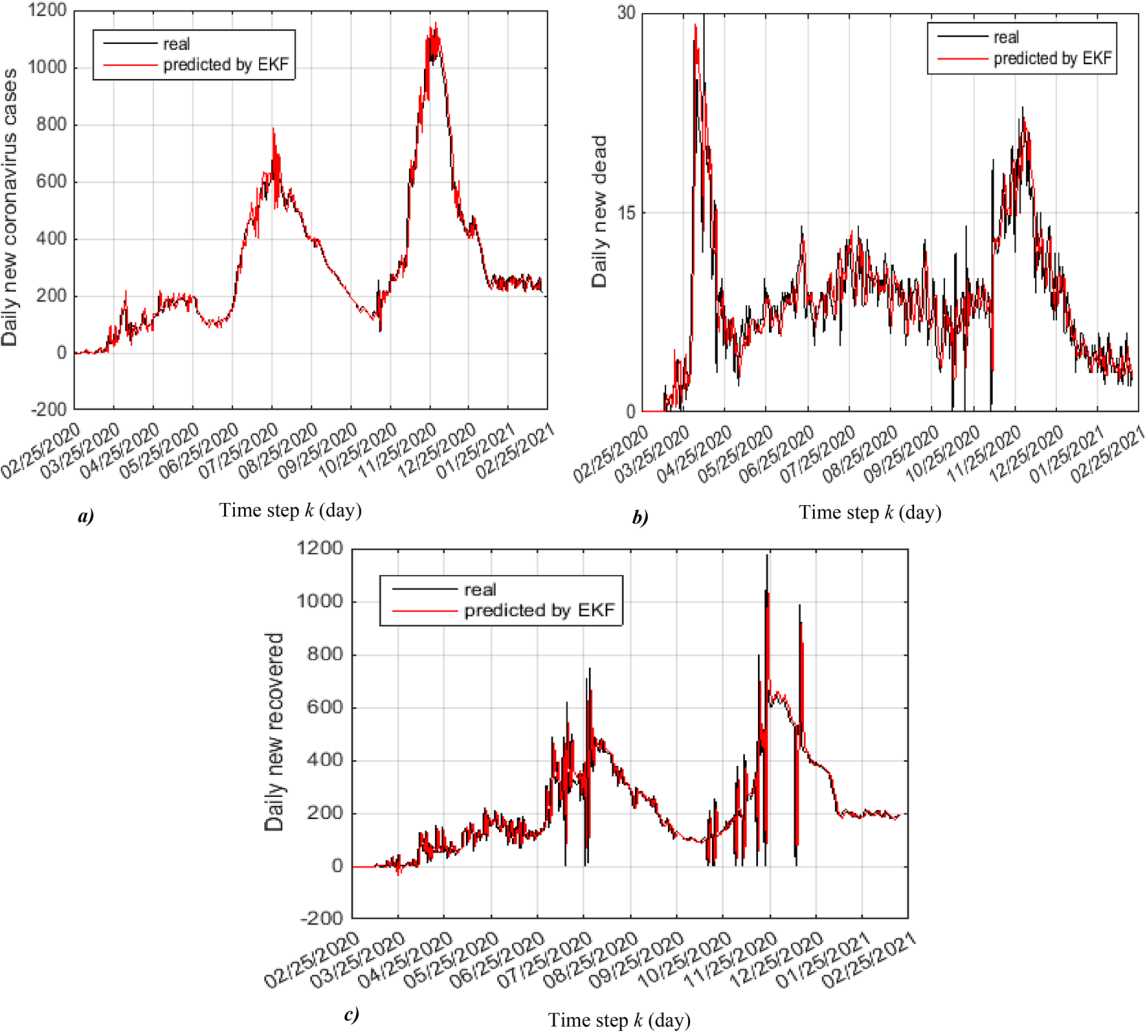
Real and predicted trajectories, (**a**) daily new coronavirus cases, (**b**) daily new dead and (**c**) daily new recovered.

Figure 8a, b and c show the real and predicted trajectories of daily new coronavirus cases, daily new deceased and daily new recovered, from these trajectories it shown that the EKF is correctly predicted these daily new quantities.

**Figure 9:**
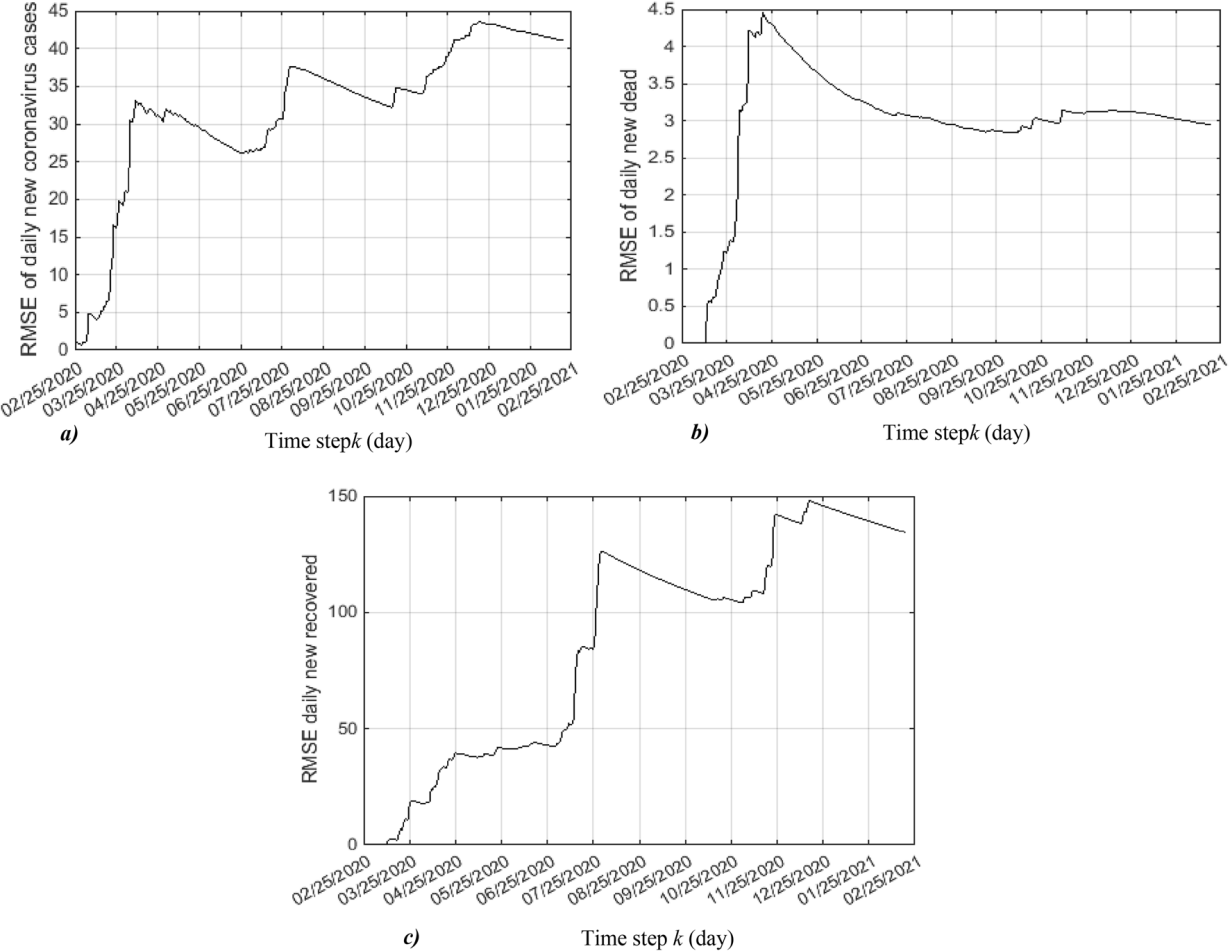
RMS error of: (**a**) daily new coronavirus cases, (**b**) daily new dead and (**c**) daily new recovered.

The good results obtained by the application of the EKF on the coronavirus evolution using SIRD model are demonstrated by the smaller RMSEs (Root mean square errors) of daily new coronavirus cases, daily new deceased and daily new recovered, illustrated in Fig. 9a, b and c.

These RMSEs are obtained from 100 Monte Carlo runs given by the equation:

35 $$RMSE\left( {x\left( j \right)} \right) = \sqrt {\frac{{\mathop \sum \nolimits_{k = 1}^{j} \left( {x_{real} \left( k \right) - x_{predicted} \left( k \right)} \right)}}{j}} j = 1, \ldots \ldots ..$$

## Conclusion

To track and predict the spread of coronavirus pandemic, we investigated and analysed the outbreak of this Covid-19 disease in Algeria, to help the government and the health ministry take new measures and future decisions to deal with this coronavirus pandemic.

For this, we supposed that the coronavirus epidemic is a target modelled by a nonlinear SIRD model and we apply the engineering technique of target tracking (an EKF algorithm) on the coronavirus spreading to predict daily all parameters i.e., susceptible (S), infected (I), recovered (R) and deceased (D).

The novelty of this work is summed up in two points: the daily updating of the model parameters and the application of the extended Kalman filter on this model, which makes the prediction results more precise and the method more reliable.

The results showed that according to the data provided by the Ministry of Algerian health and the WHO, from February 25, 2020 to February 13, 2021, the EKF algorithm is successfully predicted the daily coronavirus spreading.

## Supplementary Information

Supplementary Information.

## Data Availability

The datasets generated and/or analysed during the current study are available in the [Database Algeria Covid19] https://laig.univ-guelma.dz/sites/laig.univ-guelma.dz/files/Database_%20Algeria_Covid19.xlsx or https://laig.univ-guelma.dz/fr/node/209.
